# Embedded researchers in public health: a critical assessment

**DOI:** 10.1177/17579139231223711

**Published:** 2024-01-26

**Authors:** J Woodall, AJ Potts, SSJ Brown

**Affiliations:** School of Health, Leeds Beckett University, Portland Building, Leeds LS1 3HE, UK; Carnegie School of Sport, Leeds Beckett University, Leeds, UK; School of Health, Leeds Beckett University, Leeds, UK

**Keywords:** embedded researcher, research, evidence-based

The notion of evidence-informed policy and practice in public health is a laudable aim, but many barriers exist to generating and interpreting research evidence to influence health outcomes and reduce health inequalities.^1^ One potential avenue to consider is Embedded Researchers (ERs). This short report seeks to act as an introduction to the ER role and open up further discussion and dialogue on their utility in public health contexts.

The origins of the ER role can be traced to anthropological and sociological traditions, but in recent times there has been a rapid growth in use of ER across healthcare and public health contexts.^1–3^ Indeed, a recent review suggested that most published papers exploring ER approaches in health contexts were relatively new, with few studies dating before 2017.^4^ ERs are, nevertheless, becoming more commonplace, with a range of examples being published in public health.^1,3^ Given the increasing popularity of the ER role because of its potential to bridge the gap between academia and practice, and by extension create research-informed public health decision-making by elected members in local government, it is important that those working in academia and public health policy and practice understand this role more comprehensively.

Similar to other methodological strategies, ER has been defined in various ways by various commentators. Most definitions, however, acknowledge that ERs are university-employed and undertake explicit research roles within host organisations,^3^ such as local authority public health departments or voluntary and community sector organisations. Given the diversity of organisations that ERs could be found, Ward et al.^4^ have suggested that ER models can be complex and require some nuanced understanding of context. This suggests a one-size-fits-all approach to ER would be relatively ineffectual, but some health scholars have put forward step-by-step approaches to undertaking ER.^5^ Perhaps more useful are the published accounts of ERs that offer more practical recommendations for the consideration of ER, which offers flexible guidance for both the host organisations’ implementation team and the ERs themselves.^3^

ERs have gained popularity for a myriad of reasons. Effective knowledge exchange is cited as a primary benefit, as ERs are often able to align research rigour, situational context and independence with practical application for policy and practice in the host organisation.^6^ This is often due to the co-produced nature of the ER model where stakeholders integrated in practice settings have an impact on the outcomes and process of the research, thereby enhancing the utility of it.^2^ In this regard, ERs can enhance capacity within the host organisation and provide opportunities for practitioners to develop their research skills and interests through support from and observing the ER.^7^ This could have a positive impact on the culture of the organisation and its relationship to evidence and evidence-based practice. As already noted, public health decision-making in local government is driven by elected members who have suggested elsewhere that having good evidence to hand (perhaps through ER models) would improve their ability to inform local public health strategies and policies.^1^

Notwithstanding the positive benefits, the ER model does face criticism. The duality of the role can be problematic, both conceptually and practically, as ERs must often negotiate two different cultures, ways of working, and often competing and conflicting agendas, as well as more functional issues such as managing two email addresses and affiliations.^3^ Ironically, despite being in two organisations, the role can also be isolating, which is why clear supervision and support processes are crucial to ensuring success in the role. In addition, ERs can be recruited with a wealth of academic skills, but fewer ‘real world’ experiences of the challenges associated with putting evidence into practice in political contexts and may feel ill-equipped to deal with the presenting challenges that may require more experience or ‘soft skills’.^8^ Specific communication and relationship building skills may not always be fully developed in ERs, for example.^9^

An issue that is sometimes seen in local government,^1^ is the challenge for ERs to undertake their work expediently – and perhaps in a rushed way – in order to meet the needs of decision-makers who require evidence to inform policy in an almost instantaneous fashion. This perhaps reiterates the cultural tensions that ERs can face. Indeed, it is often the case that policy decisions can frequently be underpinned by political timeliness (based on perceived short-term opportunities and political preferences), rather than credible research evidence.^10^ This can create significant ethical and practical challenges for ERs in public health roles who may need to compromise rigour for political timeliness. Linked to this is that ERs within host organisations are often the only resource for undertaking research, which can be frustrating and can sometimes lead to host organisations having unrealistic expectations of what an ER may be able to achieve in their practice.^9^

It is evident by the increased demand for ERs in organisations, such as local government, that this role offers some distinct appeal. However, the role needs to be carefully assessed and evaluated to ensure that the benefits outweigh the costs.^3^ The increased proximity of academia to policy and practice and *vice versa* is laudable and benefits both sectors. Furthermore, increased drives towards more evidence-informed and evidence-driven policy and practice can only be a positive for improving public health outcomes and reducing health inequalities. While there are substantial challenges of the ER approach, these issues do not seem insurmountable and could be mitigated against by drawing on the experiences of those who have been, or are, an ER. The potential concern is that in the rush to implement the ER approach, for its apparent benefits to the host organisations, issues may arise and therefore extreme care must be taken in ensuring the role maintains academic rigour and is not unduly compromised. This short paper sought to outline the current literature on ERs and has described the opportunities that such an approach brings to the public health community. Further research, dialogue and debate on the ER will only refine and develop the role further.
